# Interpregnancy weight change and perinatal outcomes: A protocol for a systematic review and meta-analysis

**DOI:** 10.1097/MD.0000000000015470

**Published:** 2019-05-17

**Authors:** Jose Alberto Martínez-Hortelano, Carlos Berlanga-Macías, Diana Patricia Pozuelo-Carrascosa, Gema Sanabria-Martínez, Raquel Poyatos-León, Vicente Martínez-Vizcaíno

**Affiliations:** a Universidad de Castilla- La Mancha, Health and Social Care Research Center; b Virgen de la Luz Hospital, Cuenca, Spain; c Universidad Autónoma de Chile, Faculty of Health Sciences. Talca, Chile.

**Keywords:** body mass index, body weight, gestational outcomes, intergestational, interpregnancy, maternal outcomes and neonatal outcomes, perinatal outcomes

## Abstract

**Background:**

Growing evidence suggests that interpregnancy weight change (IPWC) is a risk factor for perinatal outcomes, since it may increase the probability of gestational complications including gestational diabetes or cesarean delivery. Additionally, IPWC may affect neonatal outcomes increasing the prevalence of newborns small for gestational age or preterm birth. However, the association between IPWC and perinatal outcomes has not systematically synthesized thus far. This study protocol aims to provide a clear, transparent and standardized procedure for systematically reviewing the association between IPWC and perinatal outcomes.

**Methods and analysis:**

This systematic review and meta-analyses protocol is based on the preferred reporting items for systematic review and meta-analysis protocols and the Cochrane Collaboration Handbook. MEDLINE, EMBASE, the Cochrane Library, and Web of Science will be systematically searched from their inception. No limits will be defined by study design, as such different tools to assess risk of bias will be used:

(1) a critical appraisal checklist for analytical cross-sectional studies;
(2) the Newcastle–Ottawa quality assessment scale for longitudinal studies (including case–control and cohort studies); and
(3) Cochrane Collaboration's tool for clinical trials.

Odd ratios and their corresponding 95% confidence intervals will be reported to evaluate associations between IPWC and perinatal outcomes.

**Results:**

The results of this study will be published in a peer-reviewed journal.

**Conclusion:**

This systematic review and meta-analysis will systematically synthesize the evidence regarding the association between IPWC and perinatal outcomes. Data will be extracted from published articles and findings will be published in peer-reviewed journals. Ethical approval and informed consent will not be required due to the nature of the study.

**Systematic review registration:**

PROSPERO CRD42018100449.

## 1 Introduction

Approximately 30% of women of reproductive age are overweight or obese.^[1,2]^ prepregnancy body mass index (BMI) has been associated with adverse perinatal outcomes, such as gestational diabetes, hypertensive disorders, higher cesarean section rates, postpartum hemorrhage, low birth weight, macrosomia, death, and stillbirth.^[3–6]^ However, there is little or weak evidence regarding the association of changes in pre-pregnancy BMI or body weight between pregnancies and its association with some perinatal outcomes.

Interpregnancy weight change (IPWC) has been defined as the difference in weight between consecutive pregnancies, taking as reference either weight at the first prenatal visit^[7,8]^ or at delivery.^[9]^ Additionally, IPWC may be reported in different ways:

(1) by units of increase or decrease in BMI^[7]^;
(2) BMI changes based on the World Health Organization (WHO) classification^[10]^;
(3) weight change in kilograms or pounds^[11,12]^ or
(4) percentage weight change.^[13]^

Several studies have reported an association between IPWC and several maternal and newborn perinatal complications.^[7,14,15]^ An increase in IPWC has been related with a higher incidence of gestational diabetes,^[7,16,17]^ preeclampsia,^[7]^ higher rates of cesarean section,^[7,15,18]^ or a higher prevalence of large for gestational age (LGA),^[7,8]^ while a decrease in IPWC may result in a higher incidence of newborns small for gestational age (SGA).^[15]^

Several factors have been related with IPWC. For instance, gestational weight gain influences postpartum weight retention^[19,20]^ which is associated with IPWC.^[21]^ Moreover, parity is positively related with IPWC, so each pregnancy increases IPWC.^[22]^ Also sociodemographic factors such as educational level^[23]^ or ethnic group^[24]^ have been related to IPWC; although a Finnish study did not find differences in IPWC across different ethnic origins but by different geographic locations.^[25]^

There are no international recommendations on IPWC. Although many studies have reported their results using different classification criteria the risk for preeclampsia,^[26,27]^ hypertensive disorders,^[15,26]^ gestational diabetes,^[26,28]^ or cesarean delivery^[9,26]^ has been found to increase when IPWC rises 1 to 2 kg/m^2^ or more. Likewise, the risk of neonatal complications such as respiratory distress^[29]^ or SGA^[30]^ increases with a decrease of 1 kg/m^2^ or more between pregnancies.

## 2 Objectives

This systematic review and meta-analysis aimed to provide a clear, standardized and transparent methodology for conducting a systematic review and meta-analysis aimed to assess the association between IPWC and perinatal outcomes.

## 3 Methods

This systematic review and meta-analysis protocol is guided by the preferred reporting items for systematic review and meta-analysis protocols (PRISMA-P).^[31]^ The systematic review and meta-analysis will be guided by The meta-analysis of observational studies in epidemiology statement,^[32]^ the PRISMA,^[33]^ and the Cochrane Collaboration Handbook.^[34]^ This protocol has been registered in PROSPERO (Registration number: CRD42018100449).

### 3.1 Eligibility criteria

#### 3.1.1 Types of studies

Published studies examining the relationship between IPWC and perinatal outcomes. The exposure will be IPWC, as units of pre-pregnancy BMI, percentage pre-pregnancy BMI or weight change, weight change in kilograms or pounds, weight percentage of change or the WHO classification criteria changes at the start of the pregnancy. The study design will be observational (including cohort, case–control, and cross-sectional) or clinical trial studies without language restriction.

#### 3.1.2 Types of participants

The participants included will be mothers with at least a previous pregnancy and their offspring. No restrictions regarding race/ethnicity, sex, economic status, and education will be applied.

#### 3.1.3 Types of outcome measures

Maternal outcomes will be: type of delivery, gestational diabetes mellitus, hypertensive disorders, preterm delivery, and stillbirth; and newborn outcomes will be: Apgar score, birth weight, LGA, or SGA.

#### 3.1.4 Exclusion criteria

Studies that included twin pregnancies or higher-order multiples will be excluded.

### 3.2 Information sources

An electronic search will be conducted in MEDLINE (via PubMed), Web of Science, Cochrane Library, and EMBASE (via Scopus), from their inception.

### 3.3 Search strategy

The search will be conducted using Boolean operators and the following keywords: “interpregnancy,” “interdelivery,” “between pregnancies,” “successive pregnancies,” “consecutive pregnancies,” “weight change,” “gestational weight gain,” “maternal weight,” “pregnancy weight gain,” “body mass index,” and “BMI.” The search strategy for PubMed is shown in Table 1. The references of previous systematic reviews and of the selected studies will be reviewed to identify additional articles. Study records will be organized using the COEVIDENCE Reference Manager.

**Table 1:**
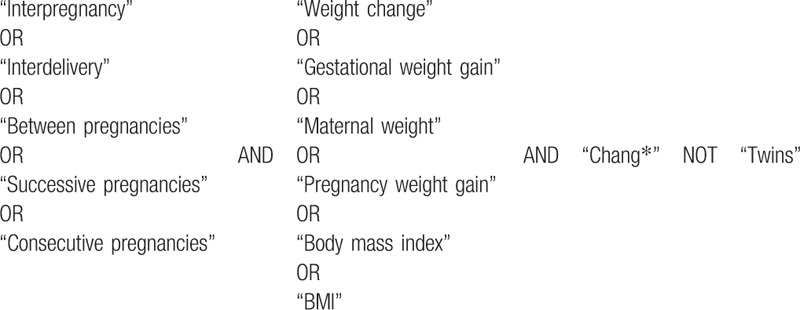
Search strategy for the MEDILINE database.

### 3.4 Data collection

#### 3.4.1 Study selection

**Figure 1:**
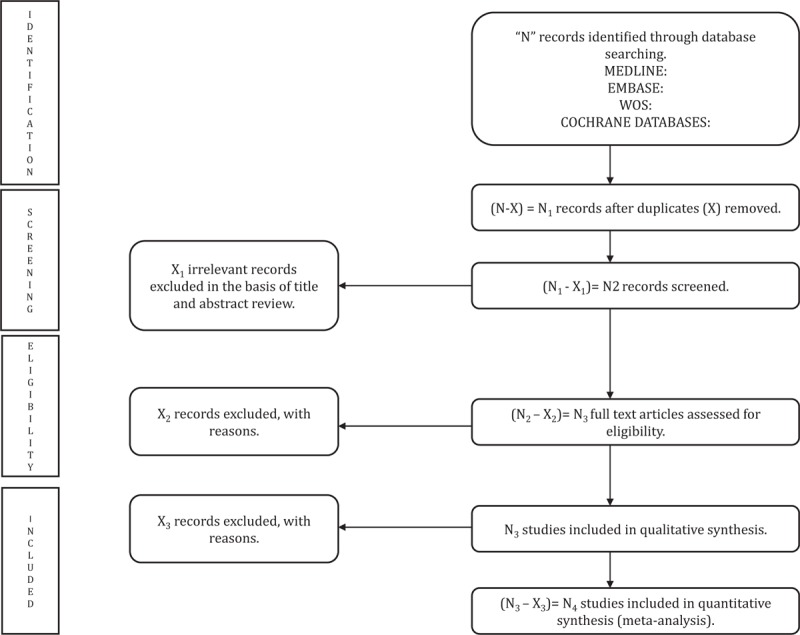
Preferred reporting items for systematic reviews and meta-analyses flow diagram of identification, screening, eligibility, and inclusion of studies.

Titles and abstracts of the retrieved articles will be independently reviewed by 2 researchers (JAM-H and DPP-C) to identify studies for this systematic review and meta-analysis. If studies do not meet the inclusion criteria, they will be excluded (Fig. 1). If the abstract does not provide enough information, the study will be selected for full-text evaluation. Two reviewers will review the included and excluded studies to verify the reasons for each decision. If consensus is not reached, a third researcher will be consulted (VM-V).

#### 3.4.2 Data extraction and management

The following data will be extracted from the selected studies:

(1) study data: author, year of publication, country, study design, sample size of mothers and children;
(2) characteristics of participants: mother's age, birth date,
(3) maternal outcomes: type of delivery, gestational diabetes mellitus, hypertensive disorders, preterm delivery or stillbirth;
(4) newborn outcomes: Apgar score, birth weight, LGA, or SGA; and
(5) adjustment variables.

### 3.5 Assessment of risk of bias

Reviewers will be blinded to the authors, title, and year of publication of the studies and quality will be independently assessed by 2 authors (JAM-H and DPP-C). Standardized checklists will be used:

(1) the Critical Appraisal Checklist for Analytical Cross Sectional Studies from The Joanna Briggs Institute will be used to assess the quality of cross-sectional studies.^[35]^ This checklist has 8 components evaluated as “Yes,” “No,” “Unclear,” and “Not applicable.” The results of this checklist will show possible bias in the design, conduct, and analysis of each study.
  Moreover,
(2) the Newcastle–Ottawa Quality assessment scale will be used to assess the quality of longitudinal studies, including case–control and cohort studies.^[36]^ This scale consists of 8 items grouped in 3 categories: (a) selection; (b) comparability; and (c) exposure in case–control studies or outcome in cohort studies. Each study can obtain 1 star for each item in the (a) selection and (c) exposure categories, and a maximum of 2 stars in the (b) comparability category.
  Finally,
(3) the Cochrane Collaboration's tool will be used for clinical trials.^[37]^ This tool evaluates 7 domains: sequence generation, allocation concealment, blinding of participants and personnel, blinding of outcome assessment, incomplete outcome data, selective outcome reporting, and “other issues.” Each item will be classified based on criteria for judging the risk of bias as: “+,” low risk of bias; “−,” high risk of bias; and “?,” unclear risk of bias.

### 3.6 Data analysis

The main characteristics of included studies and relevant information according to the aim of this systematic review and meta-analysis will be summarized in Table 2, in which the study's characteristics, population description, and relevant issues related to perinatal outcomes will be included. The reviewers will determine if meta-analysis is feasible after data extraction. At least 5 studies will be sufficient to conduct meta-analysis, if there will enough studies a narrative synthesis will be conducted. After summarizing data, a meta-analysis will be conducted using STATA V.14 software to compute pooled effect size (ES) estimates with 95% CI.

**Table 2:**
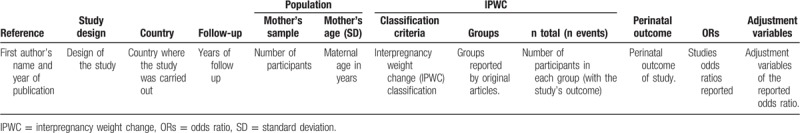
Characteristics of studies included in the systematic review and meta-analysis.

Adjusted and unadjusted results reported by original articles will be compared. The “no weight change” category will be used as the reference category, compared with the loss or increase weight categories reported by original articles, although they reported different classifications of IPWC. The heterogeneity of results across studies will be evaluated by using the *I*^2^ statistic that could be considered as: not important (0% to 40%); moderate (30% to 60%); substantial (50% to 90%), and considerable (75% to 100%), the corresponding *P*-values will be also considered.^[38]^ The pooled estimate of ES and 95% CI; will be computed using the Mantel–Haenszel fixed effects when *I*^2^ lower than 50%^[39]^ and the DerSimonian and Laird random-effects methods^[40]^ will be used with when the *I*^2^ is higher than 50%.

#### 3.6.1 Subgroup analyses and meta-regression

Subgroup analyses and meta-regression across variables that could cause heterogeneity will be conducted:

(1) study design (cross-sectional, cohort, or clinical trial studies);
(2) country;
(3) mother's characteristics: age, pre-pregnancy BMI;
(4) IPWC classification;
(5) type of postpartum intervention to manage weight such as: physical activity, diet, or another lifestyle change; and
(6) quality assessment.

#### 3.6.2 Sensitivity analysis

Included studies will be removed one by one from the pooled estimates in order to test whether the results could have been influenced by a single study.

### 3.7 Publication bias

Additionally, publication bias will be assessed using a funnel plot, according to the method proposed by Egger.^[41]^

Finally, a systematic review with descriptive analysis will be conducted if a meta-analysis is not possible due to a lack of quantitative information.

### 3.8 Evidence evaluation

Grading of recommendations, assessment, development, and evaluation (GRADE) system will be used to assess the quality of evidence of outcomes according to 4 categories: high, moderate, low, and very low. GRADE considers the elements of quality, consistency, directness, and ES.^[42]^

The results of these quality evaluations will be compared and discrepancies will be discussed. A third researcher will be asked when an agreement cannot be reached (VM-V).

## 4 Discussion

A recent meta-analysis evaluated the association between interpregnancy BMI change and pregnancy outcomes, although it included a few studies for each perinatal outcome, it excluded studies that provided IPWC data other than change in pre-pregnancy BMI in kg/m^2^.^[43]^ Furthermore, this meta-analysis excluded some perinatal outcomes because there was a lack of relevant data or low-quality studies. Additionally, more studies regarding this association have been published recently.^[28,44]^ The previous meta-analysis reported that gaining weight between pregnancies increases the risk of developing gestational diabetes mellitus, cesarean section, and LGA, as well as reducing rates of SGA.^[43]^ However, they excluded important perinatal outcomes such as preeclampsia and preterm birth. Therefore, a systematic review and, if it is possible, a meta-analysis will be conducted to highlight the influence of IPWC in perinatal outcomes. For this purpose, the protocol of this systematic review provides a clear way for extraction and synthesizing the relevant information.

Thus, if the association between higher IPWC and the increase in adverse outcomes is confirmed in the proposed study, it could support the necessity of implementing lifestyle-based interventions during the interpregnancy period, to manage IPWC in order to avoid postpartum weight retention as an important risk factor for long-term maternal obesity.^[45,46]^ Among the strategies that have been suggested to prevent higher IPWC, breastfeeding promotion,^[47]^ and structured diet and physical activity programs^[48]^ are the most noteworthy.

Because of the design variability in the included studies, 3 tools for quality assessment will be used:

(1) a Critical Appraisal Checklist for Analytical Cross Sectional Studies from The Joanna Briggs Institute for cross-sectional studies^[35]^;
(2) the Newcastle–Ottawa quality assessment scale for longitudinal studies (including case–control and cohort studies)^[36]^; and
(3) Cochrane Collaboration's tool for clinical trials.^[37]^

Furthermore, possible sources of heterogeneity, such as study design, geographical location, IPWC classification, and sample characteristics (maternal age) will be considered in this study. Moreover, random-effect meta-regressions will be used to evaluate whether these variables affect heterogeneity.^[49]^ Sensitivity and subgroup analyses will be conducted to determine sources of heterogeneity. For these reasons, there are few exclusion criteria, as such; additional analysis will be conducted in order to highlight the relevance of particular characteristics in the findings.

Potential limitations are inherent to systematic reviews and meta-analyses: publication bias, information bias, poor statically analyses, poor methodological quality, and inadequate reporting of methods and findings of the included studies. Additionally, the lack of homogeneous classification criteria of IPWC may limit comparability. These limitations should be taken into account in order to properly summarize and analyzes the information available in the manuscripts included.

In conclusion, due to the lack of evidence about the relationship between IPWC and perinatal outcomes, it is important to conduct a systematic review and meta-analysis.

## Acknowledgments

The authors would like to thank the researchers from the Health and Social Research Center for their support during the preparation of this protocol.

## Author contributions

JAM-H, CB-M, and DPP-C designed the protocol and conceived the idea for the systematic review and meta-analyses. VM-V contributed to the study design and he is the principal investigator and guarantor. CB-M, GS-M, and RP-L reviewed and edited the article and contributed to select the quality assessment tools. Finally, VM-V gave statistical support and approved the final version of the manuscript.

**Conceptualization:** Jose Alberto Martínez-Hortelano, Diana Patricia Pozuelo-Carrascosa.

**Data curation:** Jose Alberto Martínez-Hortelano, Diana Patricia Pozuelo-Carrascosa.

**Formal analysis:** Jose Alberto Martínez-Hortelano, Diana Patricia Pozuelo-Carrascosa.

**Funding acquisition:** Diana Patricia Pozuelo-Carrascosa.

**Investigation:** Jose Alberto Martínez-Hortelano, Diana Patricia Pozuelo-Carrascosa.

**Methodology:** Jose Alberto Martínez-Hortelano, Carlos Berlanga-Macías, Diana Patricia Pozuelo-Carrascosa.

**Project administration:** Vicente Martínez-Vizcaíno.

**Resources:** Gema Sanabria-Martínez.

**Software:** Jose Alberto Martínez-Hortelano, Vicente Martínez-Vizcaíno.

**Supervision:** Gema Sanabria-Martínez, Raquel Poyatos-León, Vicente Martínez-Vizcaíno.

**Validation:** Carlos Berlanga-Macías, Vicente Martínez-Vizcaíno.

**Visualization:** Vicente Martínez-Vizcaíno.

**Writing – original draft:** Jose Alberto Martínez-Hortelano.

**Writing – review and editing:** Carlos Berlanga-Macías, Diana Patricia Pozuelo-Carrascosa, Gema Sanabria-Martínez, Raquel Poyatos-León, Vicente Martínez-Vizcaíno.
